# Increased expression of Mer tyrosine kinase in circulating dendritic cells and monocytes of lupus patients: correlations with plasma interferon activity and steroid therapy

**DOI:** 10.1186/ar4517

**Published:** 2014-03-21

**Authors:** Brendan A Hilliard, Gaetano Zizzo, Mehriban Ulas, Margaret K Linan, Jessica Schreiter, Philip L Cohen

**Affiliations:** 1 Section of Rheumatology, Department of Medicine, Temple University School of Medicine, 3322 North Broad Street, Philadelphia, PA 19140, USA; 2 Temple Autoimmunity Center, Temple University School of Medicine, 3500 North Broad Street, Philadelphia, PA 19140, USA; 3 Department of Public Health, Temple University, Philadelphia, PA 19122, USA; 4 Janssen Research and Development, LLC, Spring House, PA 19002, USA

## Abstract

**Introduction:**

The requirement for the immunoregulatory Mer tyrosine kinase (Mer) for optimal removal of apoptotic cells prompted us to look at its expression in systemic lupus erythematosus (SLE), in which apoptotic cell clearance is abnormal. We compared the levels of expression of Mer in normal human subjects and in patients with SLE.

**Methods:**

We used flow cytometry of isolated peripheral blood mononuclear cells to compare the levels of Mer on leukocyte subsets. We used a Mer-specific enzyme-linked immunosorbent assay (ELISA) to quantify soluble Mer (sMer) in plasmas.

**Results:**

Monocytes, CD1c^+^ myeloid dendritic cells (mDCs), and plasmacytoid dendritic cells (pDCs) from both normal individuals and from SLE patients expressed Mer. In both normal and SLE patients, the CD14^++^CD16^+^ subpopulation of monocytes expressed the highest levels of Mer, with somewhat lower levels on the CD14^int^CD16^+^ population. Mer levels on CD1c^+^ mDCs and pDCs, and sMer levels in blood were increased in SLE patients compared with controls. In patients, Mer levels on CD14^int^CD16^+^, CD14^++^CD16^-^ monocytes, and CD1c^+^ dendritic cells correlated positively with type I interferon (IFN-I) activity detected in blood. In SLE patients treated with corticosteroids, Mer expression on monocytes correlated with prednisone dose, CD1c^+^ myeloid dendritic cells in patients treated with prednisone had higher levels of Mer expression than those in patients not receiving prednisone.

**Conclusions:**

We found no global defect in Mer expression in lupus blood. In contrast, we observed increased levels of Mer expression in DC populations, which could represent a response to increased IFN-I in SLE patients. Enhanced Mer expression induced by corticosteroids may contribute to its beneficial effects in SLE.

## Introduction

The Tyro3, Axl, and Mer receptors (TAMRs) comprise a family of receptor tyrosine kinases [1]. They are involved in the process of apoptotic debris recognition and removal in normal animals [2,3]. Their extracellular domains contain two N-terminal immunoglobulin-like domains that are essential for binding the C-terminal part of their ligands, protein S and Gas6. These two ligands have similar protein-domain structure. The N-terminal Gla domain of the ligands can bind the phosphatidyl serine on the surface of apoptotic cells, whereas two C-terminal laminin G-type receptor domains, comprising a sex hormone-binding globulin domain, can bind TAMR expressed on the surface of phagocytic cells. Activation of TAMR by Gas6 or protein S on the surface of apoptotic debris facilitates phagocytosis of the apoptotic debris and triggers an associated suppression of proinflammatory cytokine production [4-9]. Thus, Mer may have a role in controlling immune responses to antigens exposed on apoptotic cells.

In gene-expression studies, Mer (named for its expression in monocytes, endothelium, and the reproductive system) is the most highly expressed of the TAMR in the immune system, whereas Axl is expressed at lower levels in monocytes [10] and also can be expressed in dendritic cells [9,11,12]. Tyro3, conversely, is widely expressed in the central nervous system, with little expression in the immune system [1]. In human peripheral blood, little information exists about constitutive TAMR expression. Mer is expressed in a population of pulmonary macrophages isolated from bronchoalveolar lavage, and its expression is increased in smokers [13] Mer expression is also increased on monocytes and neutrophils in patients with septic shock [14].

In mice, the TAMRs are implicated in the phagocytosis of apoptotic debris in the retina, the testis, and the hematopoietic and immune systems [2]. Activation of Mer provokes changes in the cytoskeleton of phagocytes, leading to the ingestion of apoptotic debris [15]. Mer deficiency results in a defective ability to remove apoptotic cells [3], which leads to a buildup of apoptotic debris. This accumulation of apoptotic cells and debris may stimulate autoreactivity [16].

Importantly, Mer expressed in DCs is an important factor in maintaining peripheral tolerance [7]. Mer deficiency could thus contribute to lack of tolerogenic dendritic cells and to excess apoptotic cell-derived antigen presentation.

A cardinal feature of human systemic lupus erythematosus (SLE) is the presence of autoantibodies against nuclear antigens. Because humans with SLE have been found to have defective clearance of apoptotic cells [17-20], it has been proposed that the nuclear antigens contained in such debris may serve as immunogens for anti-nuclear antibody immune responses [21].

Because clearance of apoptotic debris is impaired in SLE, we wondered whether TAMR expression might be diminished in this disorder. In SLE, perturbations in the levels of TAMR ligands Gas6 and protein S and soluble TAMR are associated with disease activity [22-25], indicating that TAMR may be involved in disease progression. To examine the hypothesis that TAMRs are deficient in patients with lupus, we first tested and identified antibodies that could reliably detect TAMR. We were able to identify antibodies that could reliably detect Mer and Tyro3; however, we were unable to find an antibody that could specifically recognize Axl. We used flow cytometry to examine the expression of Mer and Tyro3 on peripheral blood leukocytes. We found that Mer was expressed on human monocytes, especially on the CD14^++^CD16^+^ subpopulation, and also on CD1c^+^ myeloid DCs, and at lesser levels on pDCs. When myeloid and plasmacytoid dendritic cells from SLE patients were compared with those from normal healthy subjects, we found that expression of Mer was significantly increased in lupus patients compared with normal subjects. This increase in expression could result from inflammatory stimulation, and indeed, Mer expression correlated with IFN-I activity in patient plasma.

## Materials and methods

### Subject recruitment

Patients and healthy controls consented to donate blood in accordance with a protocol approved by the Temple University Institutional Review Board. Normal subjects were recruited from students and workers at the Temple Health Sciences Campus. Only healthy subjects reporting no chronic illness or acute infections were recruited. SLE patients were from the Rheumatology Clinic at Temple University Hospital. Patients were diagnosed with lupus and fulfilled at least four American College of Rheumatology criteria (1997 Revision). The research protocol and consent forms were approved by the Temple University Institutional Review Board.

### Flow cytometry

After informed consent was obtained, venous blood was drawn from patients and normal subjects into EDTA-containing vacutainers (Becton Dickinson, Franklin Lakes, NJ). Peripheral blood mononuclear cells (PBMCs) were purified from blood by using Ficoll/Hypaque sedimentation to remove red blood cells and granulocytes. Then 0.5 × 10^6^ cells were stained with fluorescent antibodies for analysis with flow cytometry. Antibodies used in the study and specific for Mer (clone 125518) and Tyro 3 (clone 96201) were obtained from R&D (Minneapolis, MN, USA); Anti–CD1c (clone L161), -CD11c (clone BU15), -CD14 (clone M5E2), -CD42b (clone HIP1), -CD56 (clone HCD56), -CD 123 (clone 6H6), and -FcERI (clone AER-37) were obtained from Biolegend, San Diego, CA, USA; Anti-CD4 (clone RPA-T4) was obtained from eBiosciences (San Diego, CA, USA), and Anti-CD3 (clone UCHT1), -CD16 (clone 3G8), -CD19 (clone SJ25C1), and -HLA-DR (clone G46-6), were obtained from BD Biosciences, San Jose, CA, USA.

PBMCs were blocked by using a mixture of normal IgG from human and goat (1 mg each per tube). Antibody panels were added, and the cells were incubated on ice. Cells were washed and fixed in 1% paraformaldehyde for 10 minutes, and washed and resuspended in PBS for analysis. Leukocyte subpopulations were defined by using antibodies to CD3, CD14, CD19, and CD56 to delineate T cells, monocytes, and B and NK cells, respectively. Anti-CD42b was used to exclude leukocyte-platelet aggregates, which are reported to express TAMR. Flow-cytometric data were collected on an LSRII flow cytometer (Becton Dickinson, Franklin Lakes, NJ, USA) and analyzed by using FlowJo software (Treestar, Ashland, OR, USA). To define subpopulations of monocytes, we used a five-color strategy including anti-CD16 (clone 3G8, BD Biosciences), anti-CD14, and anti-MHC II to identify subsets of monocytes, and collected the data on a FACSCanto flow cytometer (Becton-Dickinson). To define dendritic cells, we used a six-color strategy with anti-CD1c, -CD3, -CD19, -CD123, -HLADR, in addition to anti-Mer to identify CD1c^+^ myeloid DCs, the majority of myeloid dendritic cells. We used antibody-coated beads to compensate the data; however, we found that the plasmacytoid dendritic cells CD123-PE.Cy7^+^ gave values for PE (Mer) in the negative range by using this compensation; therefore, we used manual compensation PE-Cy7-PE to adjust the PE-Cy7^hi^ cell population to match the PE fluorescence values of the PE-Cy7-negative cells in a cell sample that contained no Mer-labeled antibody.

### sMer ELISA

The total Mer ELISA kit duoset from R&D was used to quantify Mer in plasmas from lupus patients and normal controls. Conventional methods were used to carry out the ELISA. The monoclonal capture antibody at a concentration of 1.6 μg/ml was used to coat high-binding 96-well plates. Plates were blocked with 1% BSA. Plasmas were diluted 1:4 in PBS containing 1% BSA for use in the assay. The polyclonal biotinylated detection antibody was used at a concentration of 200 ng/ml. Horseradish peroxidase conjugated to streptavidin (R&D) was used with TMB substrate to detect the antigen. The ELISA could detect the extracellular portion of Mer in a Mer-Human Fc fusion protein.

### Interferon type 1 (IFN-I) activity assay

This assay measures IFN-I activity by using an interferon-sensitive reporter element linked to an alkaline phosphatase gene. Hek Blue ISRE-SEAP cells (InvivoGen, San Diego, CA, USA) were plated at 50,000 cells per well in 200 μl media and incubated overnight at 37°C. The next day, supernatants were removed from cells and replaced with 100 μl of a 50% plasma/media dilution. Cells were incubated overnight with plasma at 37°C. Next day, 40 μl of supernatant from every well was added to 160 μl of AP substrate and allowed to incubate for 20 minutes. The plate was read at 650-nm absorbance by using a spectrophotometer.

### Statistics

The Welch *t* test was used for statistical comparison of TAMRs between SLE patients and normal controls for data that showed gaussian distribution and unequal variances. Spearman Rank correlation test was used with data that showed non-gaussian distribution. Graphpad Prism (GraphPad Software, Inc., La Jolla, CA) was used for the comparison and correlative analyses. For all tests, significance was defined as *P* < 0.05.

### SLE disease activity index (SLEDAI) estimation

The SLEDAI Selena modification was used for scoring [26]. The score was estimated by examining the patient chart and the doctor’s comments of the day of the patient blood draw. The patient was considered to be anti-double-stranded DNA positive on the day of the visit if the patient had positive serology in the past.

## Results

We recruited a total of 48 lupus patients and 35 healthy individuals for our study. These subjects were used in two groups. The first cohort was used to study the expression of Mer and Tyro3 in leukocyte subpopulations (Group 1), and the second, to study the expression of Mer on monocyte subsets and dendritic cell subsets (Group 2). The clinical characteristics of the groups are given in Table 1. It should be noted that the control and lupus cohorts are not well matched for gender and ethnicity. We examined the effect of gender on the expression of Mer, and the results are described later.

**Table 1:** Healthy normal and SLE patient characteristics

	**Group 1**		**Group 2**	
**Demographic**	**Lupus**	**Normal**	**Lupus**	**Normal**
Age (years)	37.87 ± 10.23	38.41 ± 13.82	46.1 ± 14.0	40.5 ± 12.4
Sex F:M	21:1	5:9	26:3	8:7
**Ethnicity**				
African-American	15	3	19	7
Hispanic	7	0	5	0
Caucasian	0	11	6	5
Asian	0	0	0	4
**Parameter**				
Anti-dsDNA	10/22		18/29	
Anti-Sm	8/22		16/29	
Anti-Ro	6/22		12/29	
Anti-La	2/22		1/29	
Anti-cardiolipin	6/22		5/29	
Lupus anticoagulant	2/22		5/29	
Anti-B_2_ glycoprotein I	1/22		6/29	
Anti–phospholipid	3/22		3/29	
C3 low (<90)	6/22		7/29	
C4 low (<16)	5/22		8/29	
SLEDAI mean ± StDev	3.17 ± 3.13		5.6 ± 3.4	

### Expression of TAMRs on peripheral blood mononuclear cells of normal individuals

**Figure 1:**
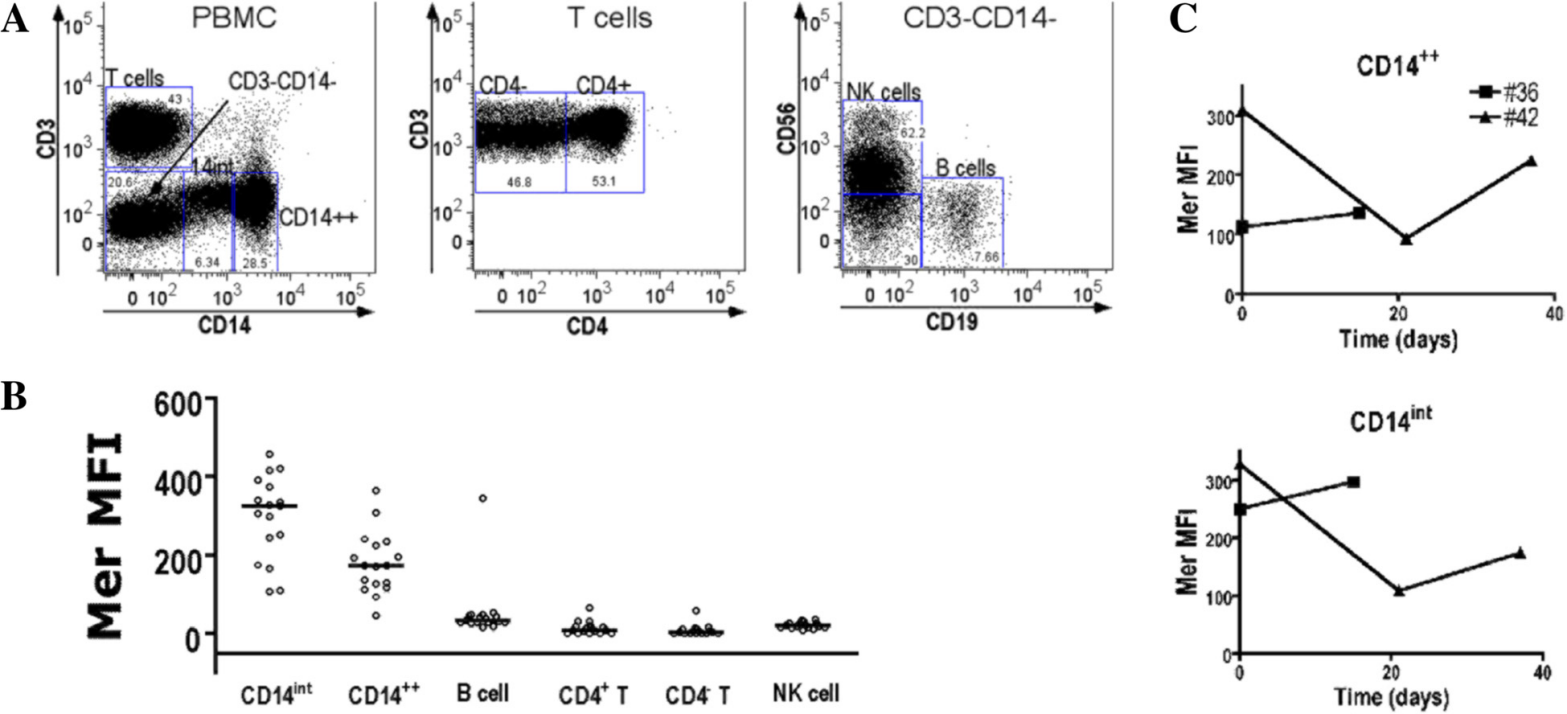
**Flow-cytometric analysis of leukocyte subsets from the peripheral blood of normal individuals. (A)** Gating strategy for identifying leukocyte subsets. **(B)** Mer expression on the surface of leukocyte subsets. MFI represents the mean fluorescence intensity obtained with the anti-Mer antibody. The data are from a group of normal healthy individuals (*n* = 14). **(C)** Mer expression varies over time. The expression of Mer on the CD14^int^ population and the CD14^++^ monocytes was examined in two normal individuals at different time points.

In initial experiments, phycoerythrin-conjugated anti-Mer and anti-Tyro3 antibodies were identified that gave positive staining on tumor cell lines known to express Mer (THP-1) and Tyro3 (K562) (see Additional file 1: Figure S1). With these antibodies, we examined the expression of the TAMR on human peripheral blood cells with multicolor flow cytometry. We included in our panel of antibodies monoclonal antibodies to recognize B (CD19), T (CD3), and natural killer (CD56) lymphocytes, and monocytes (CD14). These antibodies were used in combination to identify T, B, NK cells, monocytes with high CD14 expression, and cells expressing intermediate levels of CD14. Subset population discrimination is shown in Figure 1A. The relative levels of expression of Mer and Tyro3 on the leukocyte subsets were examined by using anti-Mer and anti-Tyro3 monoclonal antibodies. Highest expression of Mer was observed on monocytes, with minimal expression on lymphocytes. (Figure 1B; Additional file 1: Figure S2). We examined one normal subject at two different time points and another normal subject at three different time points, and observed some variation in expression over time (Figure 1C).

**Figure 2:**
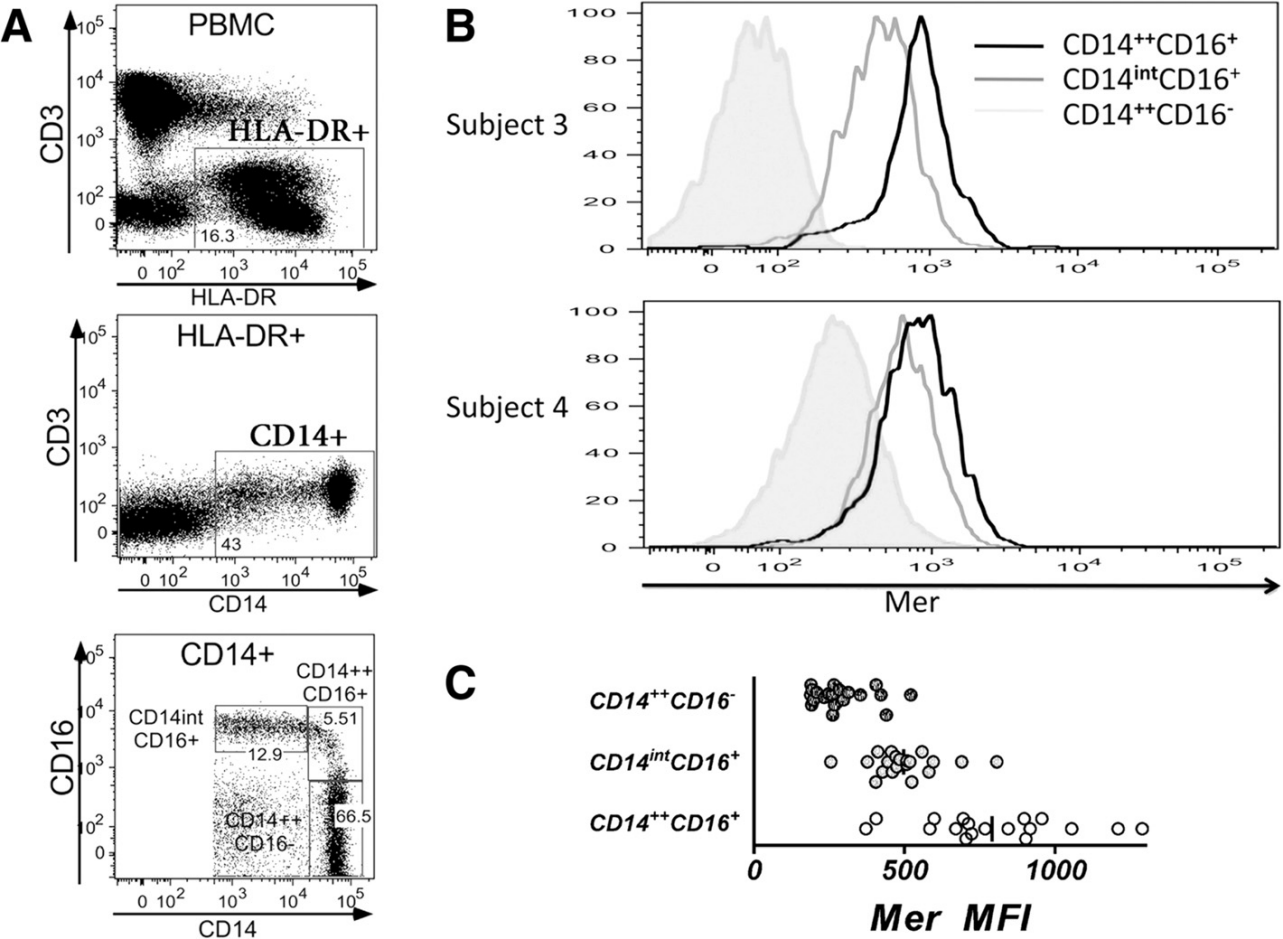
**Expression of Mer on monocyte subpopulations from the peripheral blood of normal individuals. (A)** Gating strategy for the identification of monocyte subsets. **(B)** Expression of Mer on the surface of monocyte subpopulations from the peripheral blood of two representative normal individuals. **(C)** Mean expression of Mer on monocyte subsets of normal individuals (*n* = 20).

Our initial antibody panel was designed to examine the expression of Mer and Tyro3 on the major leukocyte subsets, and we did not attempt to examine the characterized subpopulations of monocytes present in peripheral blood [27]. We observed a subset of cells expressing intermediate levels of CD14 (14^int^ in Figure 1A), and this population had higher levels of Mer compared with the monocytes expressing high levels of CD14 and myeloid dendritic cells. Because CD14 intermediate monocytes expressing CD16 are a distinct subset of monocytes with potentially important patrolling function in the vasculature [28], we wished to demonstrate that the 14^int^ population were monocytes and to look at Mer expression on the monocyte subsets. Therefore, we used another antibody panel on a second group of subjects (Group 2), by using antibodies specific for CD14, CD16, and MHC II to identify monocyte subsets (Figure 2). Interestingly, we found that within the monocyte subsets, Mer was expressed at highest levels on the population of monocytes expressing CD16 and high levels of CD14 (CD14^++^CD16^+^) monocytes [27], somewhat lower levels on the nonclassic monocytes expressing CD16, CD14^int^CD16^+^ population, and at lowest levels in the classic monocytes not expressing CD16 but expressing high levels of CD14 (CD14^++^CD16^-^) (Figure 2B, C). The classic monocytes comprise the majority of monocytes in the circulation.

**Figure 3:**
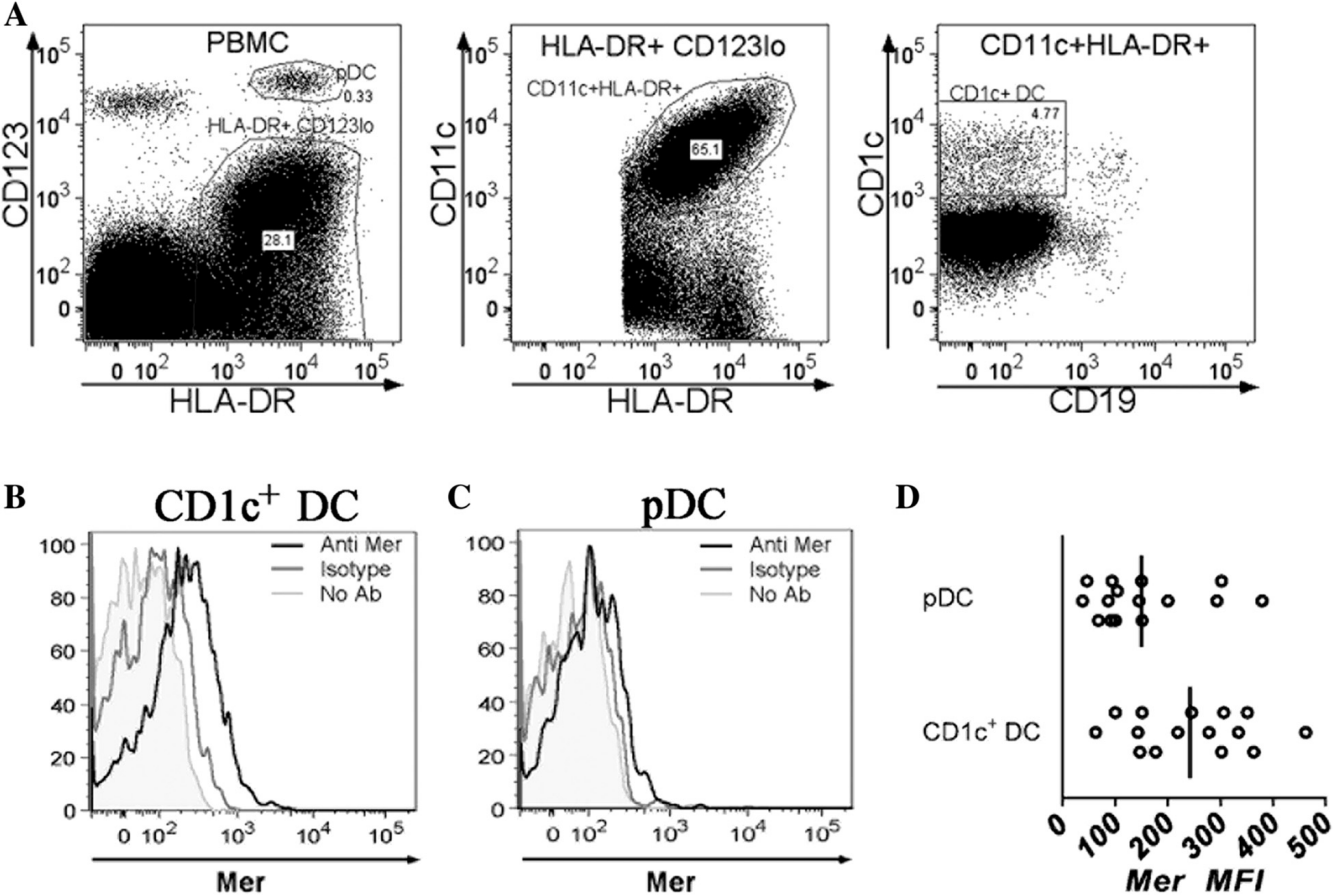
**Expression of Mer on dendritic cell populations from the peripheral blood of normal individuals. (A)** Gating strategy for the identification of dendritic cells subsets. **(B)** Expression of Mer on the surface of CD1c^+^ myeloid dendritic cells subpopulations from the peripheral blood of a representative normal individual. **(C)** Expression of Mer on the surface of pDCs on myeloid dendritic cells subpopulations from the peripheral blood of a representative normal individual. **(D)** Expression of Mer on dendritic subsets of normal individuals (*n* = 15). Horizontal bars represent mean values.

We also examined the expression of Mer on dendritic cells in the second set of subjects by using another antibody panel. Dendritic cells were identified as illustrated in Figure 3A. CD1c^+^ dendritic cells comprise the largest population of myeloid dendritic cells in normal blood [27]. These cells were identified in the HLADR^+^CD3^-^ population of cells. CD1c^+^ B cells were excluded by using CD19. Plasmacytoid dendritic cells were identified as HLADR^+^CD3^-^CD123^hi^ cells. We observed Mer expression on the surface of both CD1c^+^ myeloid DCs (mDCs) and on the surface of plasmacytoid dendritic cells (pDCs). Levels of Mer were higher on the surface of CD1c^+^ mDCs than on the surface of pDCs (Figure 3B through D).

### Expression of TAMR on peripheral blood mononuclear cells from SLE patients

**Figure 4:**
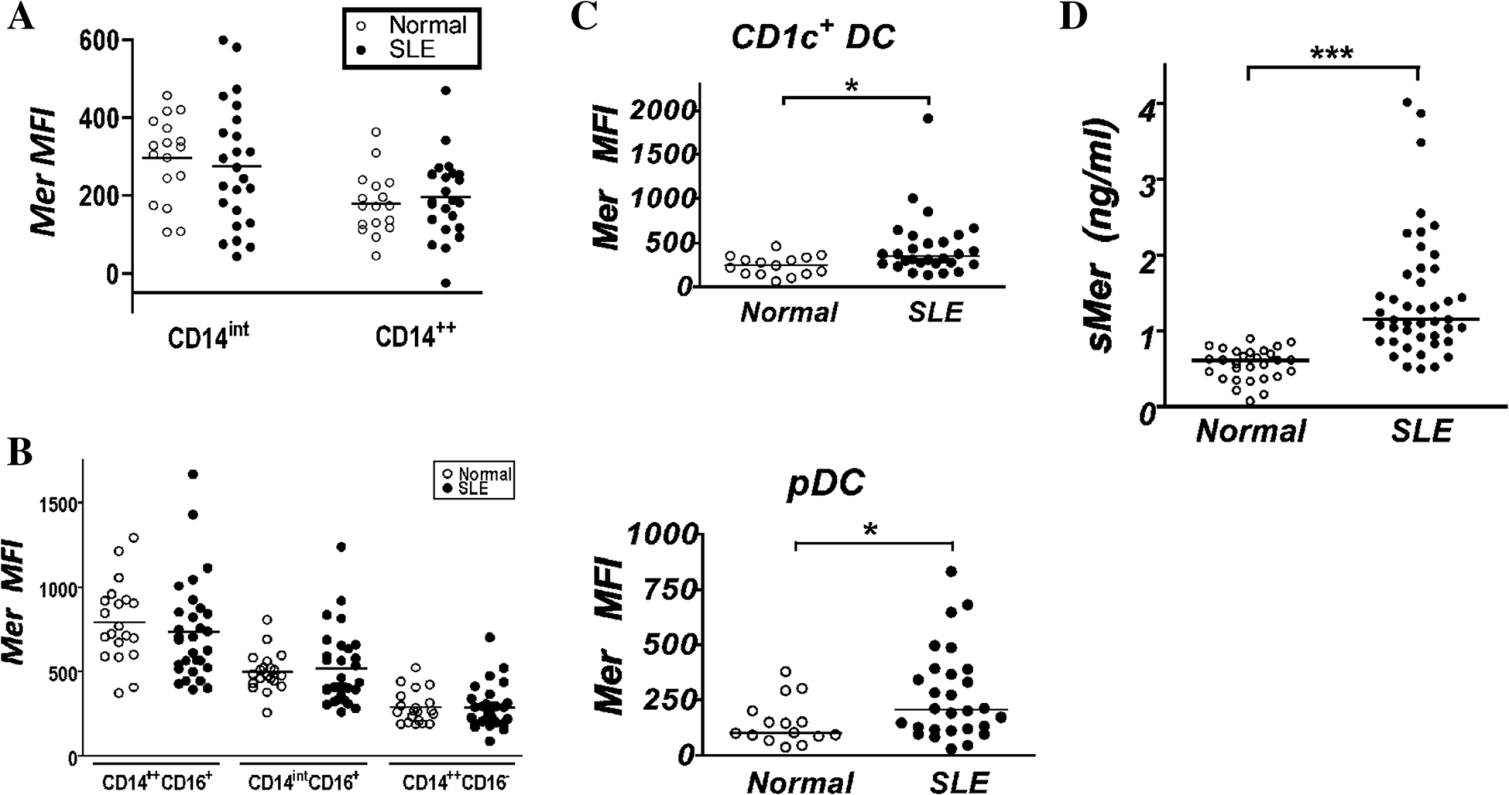
**Expression of Mer in blood from normal individuals and patients with SLE. (A)** Results from the first group of individuals for monocytes. Horizontal bars indicate means. **(B)** Results from the second group of individuals for monocytes. Horizontal bars indicate means. **(C)** Results from the second group for dendritic cell populations. Horizontal bars indicate medians, and * indicates statistical significance (Mann–Whitney, *P* < 0.05). **(D)** Quantification of soluble Mer in the plasma of lupus and healthy individuals. Horizontal bars represent median values. ***Statistical significance (Mann–Whitney, *P* < 0.001).

When we compared SLE patients with normal controls, we found no difference in expression of Mer on CD14^+^ monocyte populations in either of the two groups of subjects we tested (Figure 4A, B). Mer expression in monocytes from lupus patient blood followed the same pattern as in monocytes from healthy individuals, with highest expression in the CD14^++^CD16^+^ populations and lowest in the CD14^++^CD16^-^ populations. In CD14^++^CD16^+^ monocytes, Mer expression levels were lower in SLE compared with normal, although full statistical significance was not reached (Figure 4B). A statistically significant increase in the levels of Mer expression compared with normal subjects was observed on SLE dendritic cells.

Among DCs, a significant increase in the levels of Mer expression was found in both pDCs and in CD1c^+^ myeloid DCs from lupus patients compared with healthy control subjects (Figure 4C). The lupus myeloid DC data included one high-expression outlier; however, even if that data point were excluded from the analysis, the difference remained significant. Because the function of Mer expressed on the surface of peripheral blood leukocytes can be suppressed by the presence of sMer in the blood [29], we examined the levels of sMer in plasma samples from the normal and lupus patients. In agreement with earlier studies [23,25], increased levels of sMer were present in the blood of lupus patients compared with normal healthy individuals (Figure 4D).

Mer is generally not thought to be expressed on lymphocytes, and we found only minimal levels in our patients, yet clearly above background. When T-cell expression was examined more closely, an increase in Mer expression was found on CD4^+^ but not CD4^-^ T cells in lupus patients compared with the normal control subjects (Additional file 1: Figure S2A). The significance of this increase is uncertain.

Because our control group was not gender-matched to the predominantly female SLE patient group, we wondered whether male–female differences alone might influence Mer levels. Accordingly, we narrowed the control group to include only female subjects. Although no significant differences were found between Mer expression on monocyte subsets between lupus patients and normal healthy individuals (Additional file 1: Figure S3A), the increases in Mer expression on CD1c^+^ and pDCs in lupus patients was preserved (Additional file 1: Figure S3B). We compared the levels of Mer expressed on the monocytes and DC from female and male subjects within our control group. We did observe somewhat higher levels of Mer on the CD14^++^CD16^+^ monocyte subset in normal females compared with the SLE group (Additional file 1: Figure S3C). Also in both CD1c^+^ myeloid DC and pDC populations, Mer expression was higher in healthy men compared with healthy women (Additional file 1: Figure S3D), yet the differences failed to reach statistical significance. Thus, it is unlikely that sex differences accounted for our findings.

The possibility that racial differences were important, however, is real and will require further investigation.

**Figure 5:**
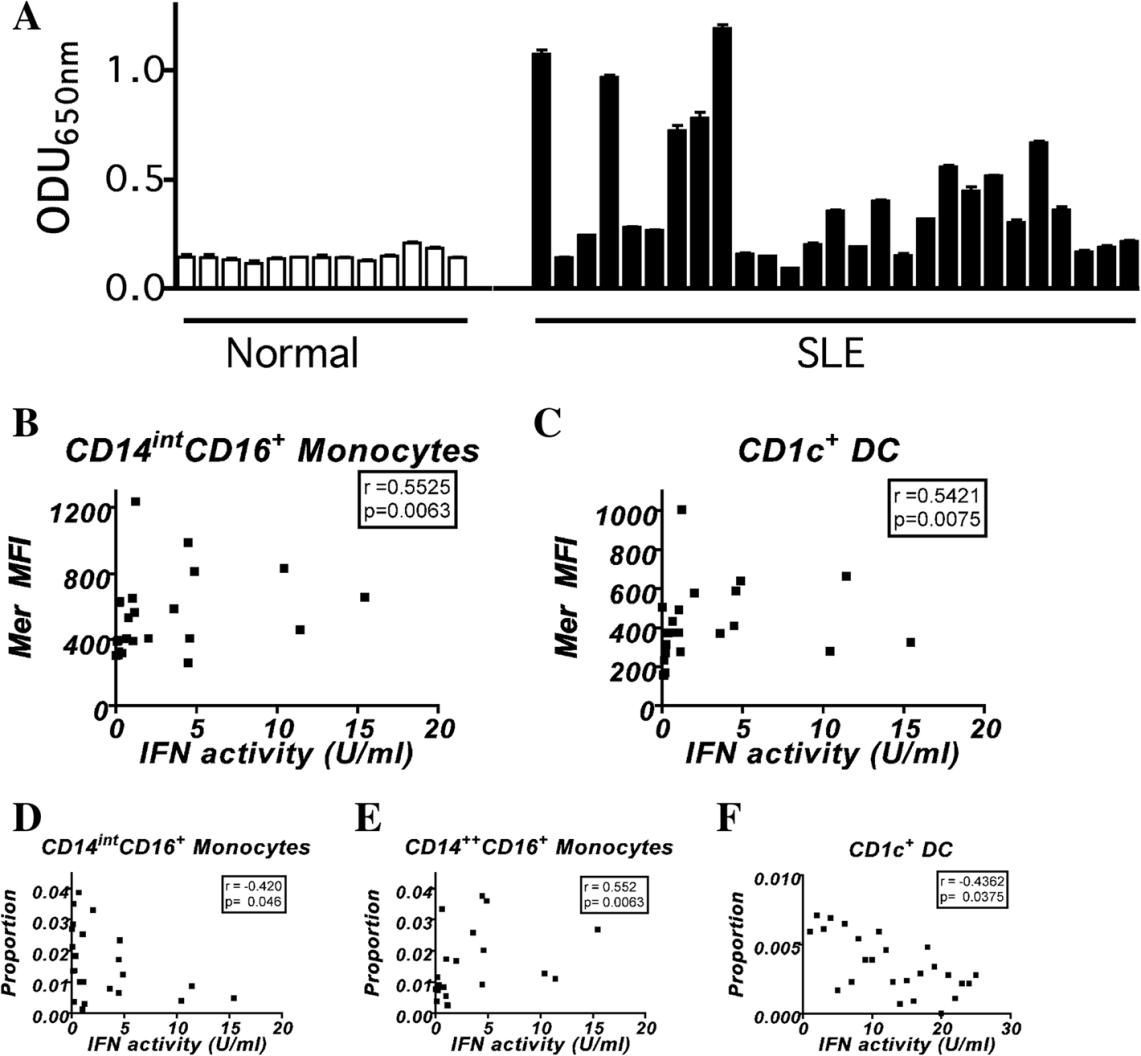
**Expression of Mer on monocytes and myeloid dendritic cells in patients with SLE correlates with IFN-I activity.** Interferon activity influences the proportion of monocytes and myeloid dendritic cells in blood. **(A)** IFN-I activity in plasmas from normal individuals and SLE patients. The optical density units obtained for plasma-induced alkaline phosphatase activity in plasmas are shown. Mer levels on **(B)** CD14^int^CD16^+^ monocyte and **(C)** CD14^++^CD16^-^ and CD1c^+^ DC subsets positively correlate with IFN-I activity. The proportions of **(D)** CD14^int^CD16^+^ monocytes, **(E)** CD14^++^CD16^-^ monocytes, and **(F)** CD1c^+^ myeloid dendritic cell subsets negatively correlate with IFN-I activity. Spearman rank correlation was used to determine significance. Values for Spearman *r* and *P* values are given for the different correlates.

Because type I interferon can activate expression of TAMR *in vitro*[9,11], and because interferon levels are related to active disease in SLE patients [30], we hypothesized that the levels of Mer might be influenced by the levels of interferon in the blood of patients with SLE. We tested the plasma of lupus patients from the same blood samples used for the flow-cytometry test for Mer expression. We used an interferon type I reporter assay with the interferon-stimulated response element that is activated by the interferon-stimulated gene factor 3 complex pathway of activation by IFN-I. We observed IFN-I activity levels above background in many of the lupus patient samples, yet in none of the normal controls (Figure 5A). Interferon activity was positively associated with Mer expression on CD14^int^CD16^+^ and CD1c^+^ myeloid dendritic cells (Figure 5B and C). Interestingly, interferon activity levels were also negatively associated with the proportion of the CD14^int^CD16^+^ monocyte subset and the CD1c^+^ DCs and positively with the proportion of the CD14^++^CD16^+^ monocyte subset (Figure 5D through F).

**Figure 6:**
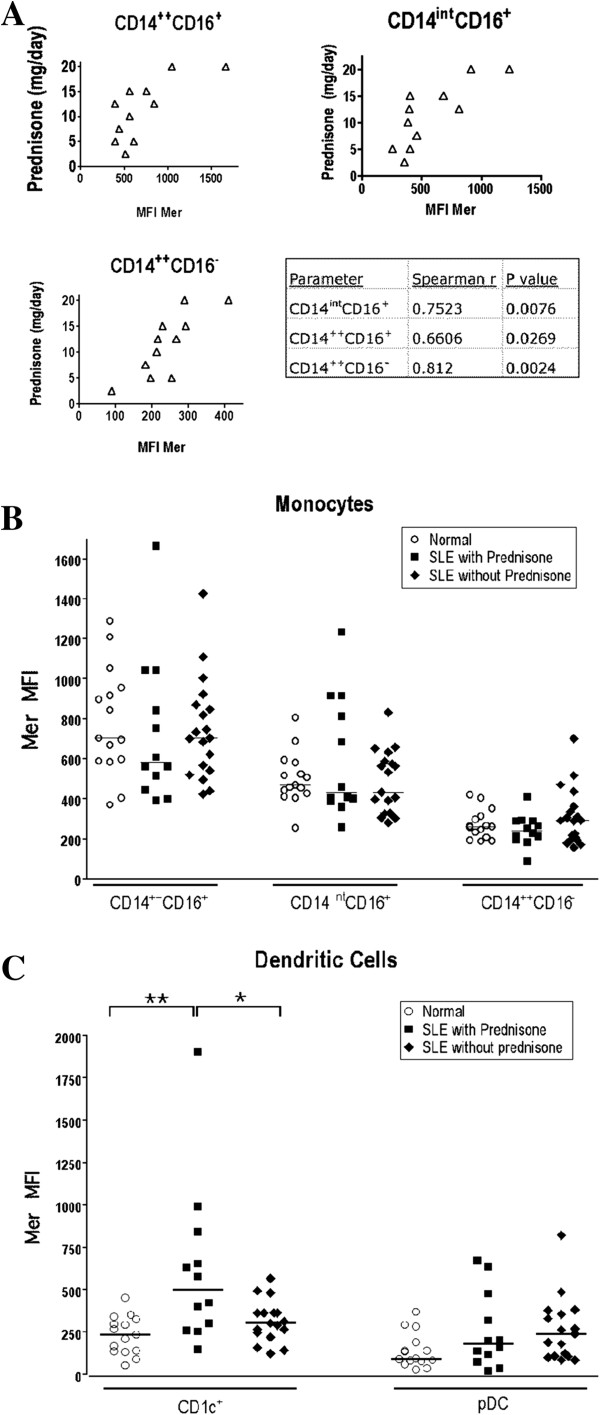
**Prednisone dose correlates with Mer expression on all three monocyte subsets in patients with SLE receiving prednisone; and prednisone treatment increases Mer expression on myeloid dendritic cells. (A)** Spearman rank correlation was used to determine the significance of correlation between Mer levels in patients receiving prednisone and dose levels of the prednisone. Values for Spearman *r* and *P* value are given for the different correlates in the Table. **(B)** Mer levels on monocytes were compared between normal healthy controls and SLE patients not treated and treated with prednisone. **(C)** Mer levels on dendritic cells were compared between normal healthy controls and SLE patients not treated and treated with prednisone. Significance was determined by using the Mann-Whitney rank correlation test. Horizontal bars indicate the median values for the groups. *P < 0.05; **P < 0.001.

We also examined associations between levels of Mer expression on the monocyte subsets and myeloid DC with various clinical parameters. Mer expression did not correlate with organ involvement, autoantibody, age, or SLEDAI score. Interestingly, the levels of Mer on the monocyte subsets in SLE patients who were being treated with prednisone were positively correlated with dose (Figure 6A). No significant difference was found in the levels of Mer expression on monocytes from those patients receiving prednisone compared with those not receiving prednisone or normal healthy control subjects (Figure 6B). However, CD1c^+^ myeloid dendritic cells in patients receiving prednisone had higher levels of Mer expression than did those in patients not receiving prednisone (Figure 6C). Levels of Mer expression on CD1c^+^ DC appeared to be higher in patients not treated with prednisone compared with normal healthy individuals; however, this difference just failed to reach significance (*P* = 0.0729)

**Figure 7:**
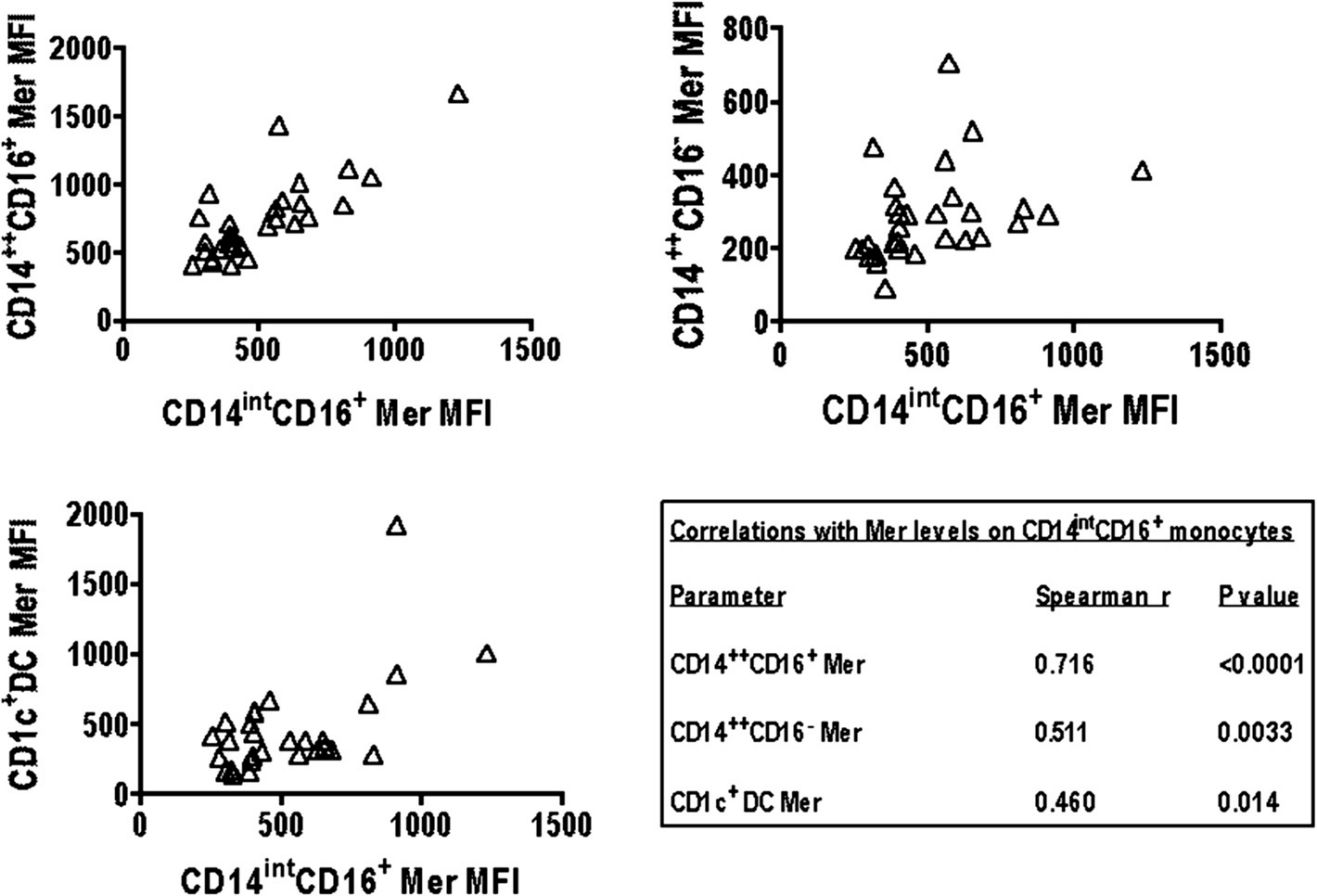
**Levels of Mer on CD14**^**int**^**CD16**^**+ **^**monocytes positively correlate with the levels of Mer in the other monocyte subgroups and on CD1c**^**+ **^**myeloid DCs.** Spearman rank correlation was used to determine significance. Values for Spearman *r* and *P* value are presented for the different subsets in the table.

**Figure 8:**
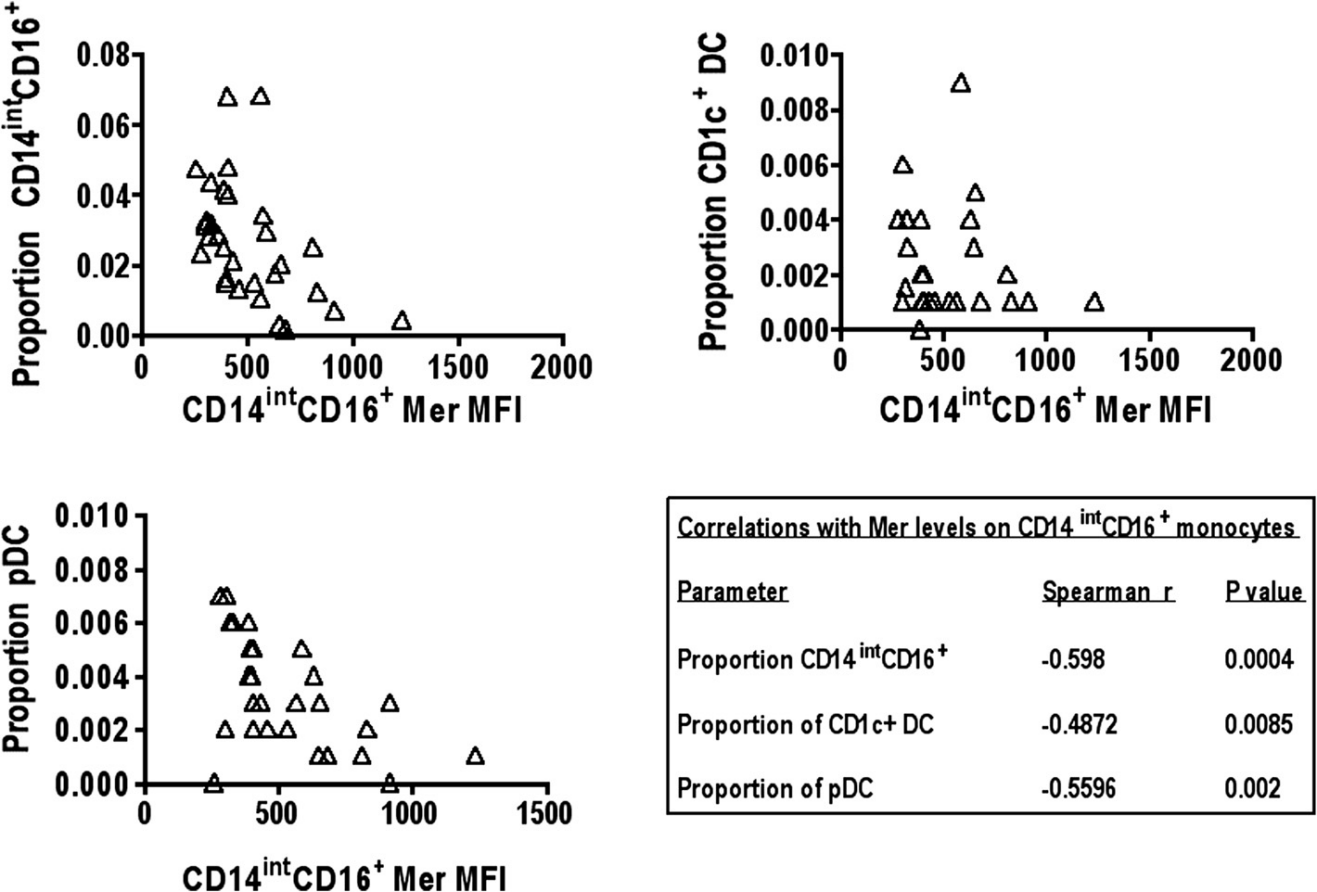
**Levels of Mer on CD14**^**int**^**CD16**^**+ **^**monocytes are negatively correlated with the proportion of CD14**^**int**^**CD16**^**+ **^**monocytes, the proportion of CD1c**^**+ **^**dendritic cells, and the proportion of pDCs in blood.** Spearman rank correlation was used to determine significance. Values for Spearman *r* and *P* values are presented for the different correlations in the table.

Of note, the Mer levels on the three monocyte subsets were highly correlated with each other and also with Mer levels on the CD1c^+^ DC population (Figure 7). The levels of Mer expression on the CD14^int^CD16^+^ subpopulation were negatively correlated with their proportion, with the proportion of the CD14^++^CD16^+^ subpopulation, and also with the proportion of both CD1c^+^ mDC and pDCs found in the blood (Figure 8). These data suggest that Mer expression on monocytes and DCs is related to their total numbers and thus to the magnitude of ongoing inflammation.

**Figure 9:**
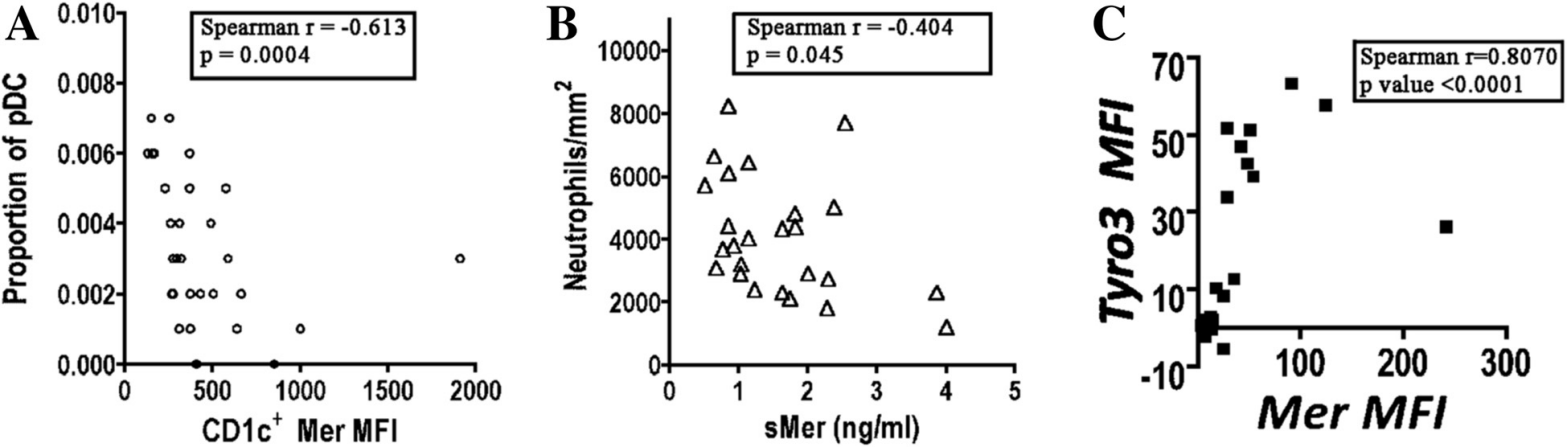
**Correlates of Mer levels in peripheral blood of lupus patients. (A)** Mer levels on CD1c^+^ dendritic cells are negatively correlated with the proportion of pDCs in lupus blood. **(B)** sMer levels in blood are negatively correlated with the numbers of neutrophils in the blood of lupus patients. **(C)** Levels of Mer on the surface of CD4^+^ T cells are positively correlated with levels of Tyro 3.

We also found a strong correlation between the levels of Mer on the CD1c^+^ mDCs with the proportion of pDCs in the blood (Figure 9A). Additionally, the proportions of both CD1c^+^ DCs and the pDCs were reduced in the SLE group compared with the normal healthy control group (Additional file 1: Figure S4).

We did not find a correlation between the levels of sMer with membrane-bound Mer on the surface of any cell type examined. However, sMer levels were negatively correlated with the numbers of neutrophils in the blood of lupus patients (Figure 9B).

Because prednisone treatment influences the levels of Mer on peripheral blood monocytes and dendritic cells, we looked at the subgroup of patients not receiving prednisone (Additional file 1: Figure S5). Notwithstanding the lower number of data points, we observed additional positive correlates of IFN-I activity with Mer expression on CD14^++^CD16^+^ monocyte populations and also notably with sMer. Increased strength of correlation (Spearman *r*) of IFN-I activity was noted, positively to the expression of Mer on the CD14^int^CD16^+^ subgroup of monocytes, and negatively to the proportion of this monocyte subpopulation present in peripheral blood. An increased strength of positive correlation of IFN-I activity also was noted with the proportion of CD14^++^CD16^+^ monocyte subpopulation,

As expected, Tyro3 was generally expressed at low levels in leukocyte subsets in peripheral blood. However, in some normal individuals and some SLE patients, Tyro3 was expressed above background levels in the CD14^int^ cells and B-cell populations (Additional file 1: Figure S2A). Surprisingly, a small but significant increase in Tyro3 expression was present on CD4^+^ T cells in lupus patients. The expression of Tyro3 was strongly associated with the expression of Mer in the CD4^+^ T lymphocytes of lupus patients (Figure 9C).

## Discussion

The present findings establish that Mer is expressed primarily on monocytes and dendritic cells in humans. Contrary to our expectations, we did not find reduced Mer expression on blood mononuclear cells from SLE patients. In contrast, the expression of Mer in certain monocytes and myeloid dendritic cells was increased, and this correlated with type I interferon levels. The cell distribution of human Mer expression parallels our findings of Mer expression in the mouse [31,32]. Normal human monocytes expressed significant levels of Mer overall, and expression was higher on CD16^+^ monocytes, especially the CD14^++^CD16^+^ subset of monocytes, with lower levels on the CD14^++^CD16^-^ subset. Mer expression is greatly increased in human macrophages matured *in vitro*[33], and specifically those differentiated along the M2 lineage [34]. The relatively low levels of Mer observed in human peripheral blood monocytes are therefore probably reflective of their relatively undifferentiated state.

Compelling evidence suggests that the three monocyte populations represent a continuous line of development in the circulation, with the CD14^++^CD16^+^ population representing a transitional phase between the least-advanced classic CD14^++^CD16^-^ monocytes and the most-mature CD14^int^CD16^+^ monocytes [35], with some characteristics of tissue macrophages [36].

However, functional differences exist between monocytes segregated into the three populations. The CD14^++^CD16^+^ subset respond well to LPS, producing more TNF-α and IL-1β than the other monocyte subsets [28], but they also produce significant amounts of the antiinflammatory cytokine IL-10 [37]. Good evidence indicates that the CD14^++^CD16^+^ monocytes can efficiently activate T cells, as they express high levels of MHC class II compared with the other subsets [38], and functionally were best at instigating a superantigen-induced T-cell response. Therefore, the higher expression of Mer on these cells suggests that it can play an important role in regulating apoptotic cell phagocytosis and any ensuing T-cell response. Based on the higher levels of Mer expression on CD14^++^CD16^+^ monocytes, a role for Mer may exist in other diseases. The CD14^++^CD16^+^ subset are expanded in patients with rheumatoid arthritis [39]. Increased numbers of the population predict the risk of cardiovascular disease in patients with chronic kidney disease [40], patients receiving hemodialysis [41], and patients selected for coronary angiography [42]. However, the expression profile of Mer in these conditions is not known.

In contrast to the CD14^++^CD16^+^ subset, the CD14^int^CD16^+^ population of monocytes respond poorly to LPS but have an enhanced reaction to viruses and nucleic acids [28]. The human CD14^int^CD16^+^ monocyte subset also have a unique patrolling function in the vasculature, as demonstrated by their ability to ”crawl” on the endothelium of mouse blood vessels, [28]. It is tempting, therefore, to propose a role in vascular endothelial repair for these specialized monocytes because of the potential role of Mer in the recognition and removal of damaged endothelial and other component cells of the vasculature that express phosphatidyl serine on their surfaces.

The increased expression of Mer in the CD16^+^ subsets of monocytes is consistent with a role for Mer in the phagocytosis of apoptotic cells, because among monocytes, the CD16^+^ monocytes preferentially phagocytose apoptotic cells [34]. The relatively high expression of Mer in the CD16^+^ cell subsets is also consistent with a role for Mer in the observed inhibition of TNF-α and IL-1β production in CD16^+^ monocytes by apoptotic neutrophils [43].

Our finding that circulating human myeloid DCs express Mer, parallels the expression of Mer on fresh splenic DCs in mice. Although Mer is not required for phagocytosis of apoptotic cells in mouse dendritic cells [31], with Axl and Tyro3 being more important for the phagocytosis of apoptotic cells by mouse splenic and bone marrow-derived dendritic cells [12], Mer functions as an important regulator of DC activation in mice [7,44]. The loss of Mer in *in vitro*-generated mouse dendritic cells led to a lack of the regulation of NF-κB activation by apoptotic cells. In a mouse model of autoimmune diabetes, loss of Mer was associated with increased numbers of pancreatic DCs and an increased ability of DCs to stimulate islet-specific T cells [45]. Therefore, a lack of Mer-dependent DC regulation could lead to an increase in T-cell autoreactivity and increased autoimmune disease.

Mer is expressed in splenic NK and NKT cells in mice, and has been shown to have functional significance in the activation of NKT cells [31]. The present data indicate that expression on circulating human NK cells is negligible. We did not find enough NKT cells in the PBMC fraction to make any conclusion regarding Mer expression. Other subsets of leukocytes, including CD8^+^ T cells and B cells, also had negligible levels of Mer expression, suggesting that Mer has no functional role in these cells under normal healthy circumstances.

We observed an increase in expression of Mer on CD1c^+^ mDCs and also in plasmacytoid DCs in lupus blood. These data suggest that no defect in Mer expression exists in lupus. The increase in Mer expression in DCs may reflect a response to an inflammatory environment in SLE akin to the activation of TAMR in response to TLR activation in monocytes [46]. The association of increased Mer expression with inflammation is also a feature of alveolar macrophages in the chronic lung inflammation seen in smokers [13] and also in monocytes in patients with septic shock [14]. *In vitro* experiments with human monocyte-derived DCs demonstrate that Axl is induced by type I interferon [9,11]. Analogously, therefore, Mer expression may also be a response to the type I interferon signature that characterizes lupus [47,48]. Thus increased Mer expression on DCs may represent a marker of IFN-I activity in lupus patients. This hypothesis is strongly supported by our results demonstrating a positive association between IFN-I activity and Mer levels on monocyte subsets and myeloid dendritic cells.

A notable negative correlation was noted between Mer expression on the CD14^int^CD16^+^ subset of monocytes with the proportions of the same CD14^int^CD16^+^ subset of monocytes, the CD1c^+^ DC, and the pDC populations. This is consistent either with a selective killing of these cell subsets, or with an increased migration of the CD16^+^ monocytes into the tissues. The former hypothesis suggests a lack of Mer-expressing phagocytes in certain lupus patients, which could be involved in the reported defect in the ability of macrophages from lupus patients to phagocytose apoptotic cells [17-20]. The latter hypothesis is supported by the established behavior of the CD14^int^CD16^+^ subset of these cells that is able to patrol the surface of the vascular endothelium and is more adherent than the CD14^++^ subpopulation of CD16^+^ and the classic CD16^-^ monocytes [28] and hence is more likely to migrate from the vasculature into tissues.

Similarly, studies focusing on the total CD16^+^ monocyte population have demonstrated the increased adherent and transmigratory ability of these monocytes. Thus, the monocyte subsets expressing most Mer are more inclined to adhere to and transmigrate through the vascular endothelium [49-51], whereupon they may then partake in the phagocytosis of apoptotic cells in tissues. Mer expressions on all three monocyte subsets are positively associated with each other and with the Mer expressed on myeloid DC, arguing for a global regulation of Mer expression on monocyte and myeloid dendritic cells.

Remarkably, we also found an inverse correlation between the expression of Mer on the CD14^int^CD16^+^ monocytes and the proportions of both the CD1c^+^ myeloid and the plasmacytoid dendritic cells. Additionally, we found that the proportions of both pDCs and CD1c^+^ myeloid dendritic cells were decreased in the SLE patient group compared with normal healthy subjects. The reduction in the numbers of pDCs has been reported [52] and may reflect recruitment from the peripheral blood to diseased tissues during disease episodes [53,54].

A role may exist for TAMR signaling in regulating autoimmunity via IFN-I signaling. In *in vitro* cultures of mouse bone marrow-derived dendritic cells, IFN-α induced Axl expression that downregulated inflammatory.

Most of the homeostatic phagocytosis that takes place in an organism most probably occurs in solid tissues rather than in peripheral blood. It is likely that the expression of Mer is increased substantially in tissue-resident macrophages and hence that these macrophages are more efficient at removing apoptotic cells compared with peripheral blood monocytes. Although we did not address the question of how much Mer is necessary to evoke a functional phagocytic or regulatory response, it seems that the differences in Mer levels between the CD16^+^ monocytes and the CD16^-^ monocytes contribute to the increased efficiency of the CD16^+^ monocytes at ingesting apoptotic neutrophils [34].

It is also possible that the increased expression of Mer on DCs in lupus patients may reflect a defect in Mer function in these cells, which could activate a compensatory pathway to upregulate Mer expression. Pertinent to this is our observation [55], and those of others [23,25], that increased levels of sMer are found in the blood of lupus patients compared with healthy control subjects. Increased levels of sAxl and sTyro3 have also been found in lupus patient blood [22,23], and all three soluble TAMRs can compete for the TAMR ligands Gas6 and protein S to block Mer function. This, through competition for ligand, can lead to a suppression of membrane-bound Mer activation [29] and a decreased function of Mer in lupus.

In mouse models of lung disease, prevention of the proteolytic cleavage of Mer led to beneficial Mer-dependent pro-phagocytic and anti-inflammatory responses [56,57]. These results in mice suggest that increasing membrane-bound Mer activation may also have a beneficial effect in SLE.

Glucocorticoids induce a Mer/protein S-dependent mechanism of apoptotic cell removal in human monocyte-derived macrophages [58]. In keeping with this *in vitro* observation, Mer expression on monocytes of our SLE patients receiving prednisone correlated strongly with the dose of corticosteroid (Figure 6A). Unexpectedly, no overall difference was noted in Mer expression on the monocytes of those lupus patients taking prednisone compared with those not given prednisone. Patients not taking prednisone at all may differ clinically from those patients who merited prednisone, even small doses. This may be reflected in the normal or slightly elevated monocyte Mer expression in the presumably inactive patients not taking corticosteroids. Yet it was clear that prednisone increased Mer expression in a dose-dependent fashion in those patients sick enough to merit prednisone treatment.

We also found that prednisone-treated lupus patients had increased Mer expression on their CD1c^+^ myeloid DCs. The apparent relation between steroid dose and monocyte and DC Mer expression parallels *in vitro* observations that glucocorticoids prominently induce Mer, together with a broader upregulation of proteins involved in the antiinflammatory phagocytic pathway specific for apoptotic cells [33,34,59]. The *in vivo* induction of Mer on monocytes, and especially dendritic cells, therefore may constitute an important part of the mechanism of immunosuppression induced by prednisone treatment.

In patients not being treated with prednisone, we found an association of sMer in blood with IFN-I activity levels. Because no direct upregulatory effect of type I IFNs was found on sMer release from *in vitro* cultures of monocytes [55], this association may depend on other disease-related factors. For example, this could reflect an increased activity in lupus blood, of specific proteases, such as ADAM10 and ADAM17 (TNF-α-converting enzyme, TACE), which are capable of enzymatically converting membrane-bound Mer to soluble Mer. Our observations also suggest that a strong homeostatic mechanism may exist for Mer expression on the surface of monocytes and dendritic cells in peripheral blood.

We found that Tyro3 and Mer are expressed on CD4^+^ T cells in some individuals with lupus. An increased Mer and Tyro3 expression on CD4^+^ cells was noted in lupus patients compared with normal control subjects. Mer levels correlated with Tyro3 levels on the surface of CD4^+^ T cells in lupus patients. The significance of this is unclear; however, it may be of interest because Mer has a prosurvival effect in the human Jurkat T-cell lymphoma [60] and also in other lymphomas overexpressing Mer [61], and therefore, it could potentially have a role in prolonging survival of autoreactive T cells in lupus. Clearly the role of TAMR in T-cell activation in inflammatory diseases deserves further study.

It will be interesting to look at other rheumatic diseases to see whether a similar regulation of Mer expression exists, and whether the Mer-expression pattern is unique to lupus.

Finally, although it is reasonable to believe that the expression of TAMR on blood leukocytes reflects the expression of Mer on immune cells in solid tissues, our study does not preclude the possibility that Mer expression is deficient in involved organs.

## Conclusions

Our data show that no defect in Mer expression is present in SLE patients. In contrast, the increased levels of Mer on the surface of myeloid and plasmacytoid DCs may reflect the enhanced IFN-I inflammatory environment in SLE, and may also represent a compensatory response to reinstate immune tolerance and effect an antiinflammatory state. Also, because corticosteroid therapy enhances Mer expression on circulating monocytes and dendritic cells, this therapy may exert beneficial effects by enhancing phagocytic capacity for apoptotic debris and restoring peripheral immune tolerance via Mer in SLE patients.

## Abbreviations

mDC: myeloid dendritic cell; Mer: Mer receptor kinase; NK: natural killer; PBMC: peripheral blood mononuclear cell; pDC: plasmacytoid dendritic cell; SLE: systemic lupus erythematosus; sMer: soluble Mer receptor; TAMR: TAM receptor family of membrane tyrosine kinase; Tyro3: tyrosine receptor kinase 3.

## Competing interests

Philip Cohen and Brendan Hilliard declare that they receive funding for other research studies from Janssen Research and Development. The remaining authors declare that they have no competing interests.

## Author’ contributions

BAH: conception, design, data collection, analysis, manuscript writing, and final approval of the manuscript. GZ: analysis, critical revision of the manuscript, MU: data collection and analysis, critical revision, and final approval of the manuscript. MKL: data analysis. critical revision, and final approval of the manuscript. JS:, data collection and analysis, critical revision, and final approval of the manuscript. PLC: conception, design, analysis, critical revision, and final approval of manuscript. All authors read and approved the final manuscript.

## Supplementary Material

Additional file 1: Figure S1 Mer and Tyro3 expression in cell lines. **(A)** Cell lines U937, phorbol myristate acetate-stimulated THP-1, and Jurkat stained with monoclonal anti-Mer-PE. **(B)** K562 cells stained with monoclonal anti-Tyro3-PE **Figure S2.** Comparison of Mer and Tyro3 expression in leukocyte populations in the blood of normal healthy subjects and SLE patients. **(A)** Expression of Mer on lymphocyte populations in normal individuals and patients with SLE. **(B)** Expression of Tyro3 on leukocyte subpopulations from normal individuals and patients with SLE. **Figure S3.** Comparison of Mer expression in normal females with SLE patients and effect of gender on expression of Mer. **(A)** Mer expression levels on monocytes from normal females and patients with SLE. **(B)** Mer-expression levels on dendritic cells from normal females and patients with SLE. **(C)** Mer expression levels on monocytes from normal female and male subjects. **(D)** Mer-expression levels on dendritic cells from normal female and male subjects. **Figure S4.** Comparison of monocyte and dendritic cells proportions in normal healthy subjects and SLE patients. **(A)** Monocyte population proportions of peripheral blood mononuclear cells are similar between SLE and normal healthy subjects. The bars represent the mean values. **(B)** CD1c^+^ and plasmacytoid dendritic cell proportions of peripheral blood mononuclear cells are reduced in patients with SLE compared with normal control subjects. **Figure S5.** Mer expression on monocytes, sMer in blood, and proportions of monocyte subsets correlate with IFN-I activity in SLE patients that do not receive prednisone. Mer levels on monocyte subsets. **(A)** CD14^++^CD16^+^, **(B)** CD14intCD16^+^, and **(C)** CD14^++^CD16^-^ and sMer levels in **(D)** plasma, positively correlate with interferon activity. Proportions of monocyte subsets correlate with IFN-I activity, negatively for **(E)** CD14intCD16^+^ monocytes, and positively for **(F)** CD14^++^CD16^-^ monocytes. Click here for file
