# Supplementary material for: Increased expression of Mer tyrosine kinase in circulating dendritic cells and monocytes of lupus patients: correlations with plasma interferon activity and steroid therapy
Source: Arthritis Res Ther. 2014 Mar 21;16(2):R76. doi: 10.1186/ar4517 (PMC4060208; doi:10.1186/ar4517)
Supplement: Additional file 1: Figure S1 — Mer and Tyro3 expression in cell lines. (A) Cell lines U937, phorbol myristate acetate-stimulated THP-1, and Jurkat stained with monoclonal anti-Mer-PE. (B) K562 cells stained with monoclonal anti-Tyro3-PE Figure S2. Comparison of Mer and Tyro3 expression in leukocyte populations in the blood of normal healthy subjects and SLE patients. (A) Expression of Mer on lymphocyte populations in normal individuals and patients with SLE. (B) Expression of Tyro3 on leukocyte subpopulations from normal individuals and patients with SLE. Figure S3. Comparison of Mer expression in normal females with SLE patients and effect of gender on expression of Mer. (A) Mer expression levels on monocytes from normal females and patients with SLE. (B) Mer-expression levels on dendritic cells from normal females and patients with SLE. (C) Mer expression levels on monocytes from normal female and male subjects. (D) Mer-expression levels on dendritic cells from normal female and male subjects. Figure S4. Comparison of monocyte and dendritic cells proportions in normal healthy subjects and SLE patients. (A) Monocyte population proportions of peripheral blood mononuclear cells are similar between SLE and normal healthy subjects. The bars represent the mean values. (B) CD1c+ and plasmacytoid dendritic cell proportions of peripheral blood mononuclear cells are reduced in patients with SLE compared with normal control subjects. Figure S5. Mer expression on monocytes, sMer in blood, and proportions of monocyte subsets correlate with IFN-I activity in SLE patients that do not receive prednisone. Mer levels on monocyte subsets. (A) CD14++CD16+, (B) CD14intCD16+, and (C) CD14++CD16- and sMer levels in (D) plasma, positively correlate with interferon activity. Proportions of monocyte subsets correlate with IFN-I activity, negatively for (E) CD14intCD16+ monocytes, and positively for (F) CD14++CD16- monocytes. [file ar4517-S1.docx]

**Additional File 1 Figure S1**


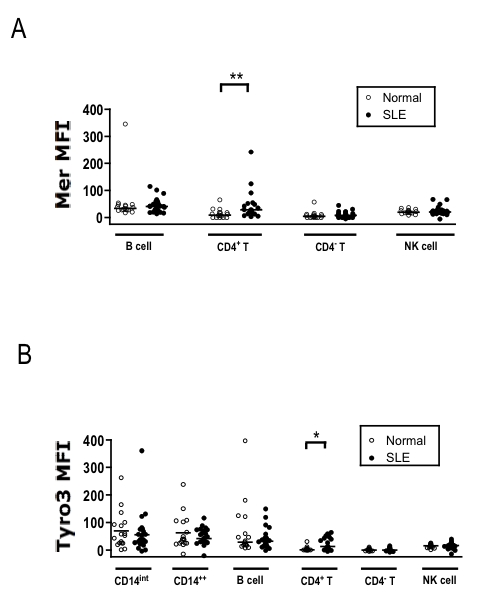


Horizontal bars represent the median while the difference between the two populations was tested using Mann-Whitney U-test. * p ≤ 0.05, ** p ≤ 0.01.

**Additional File 1 Figure S2**


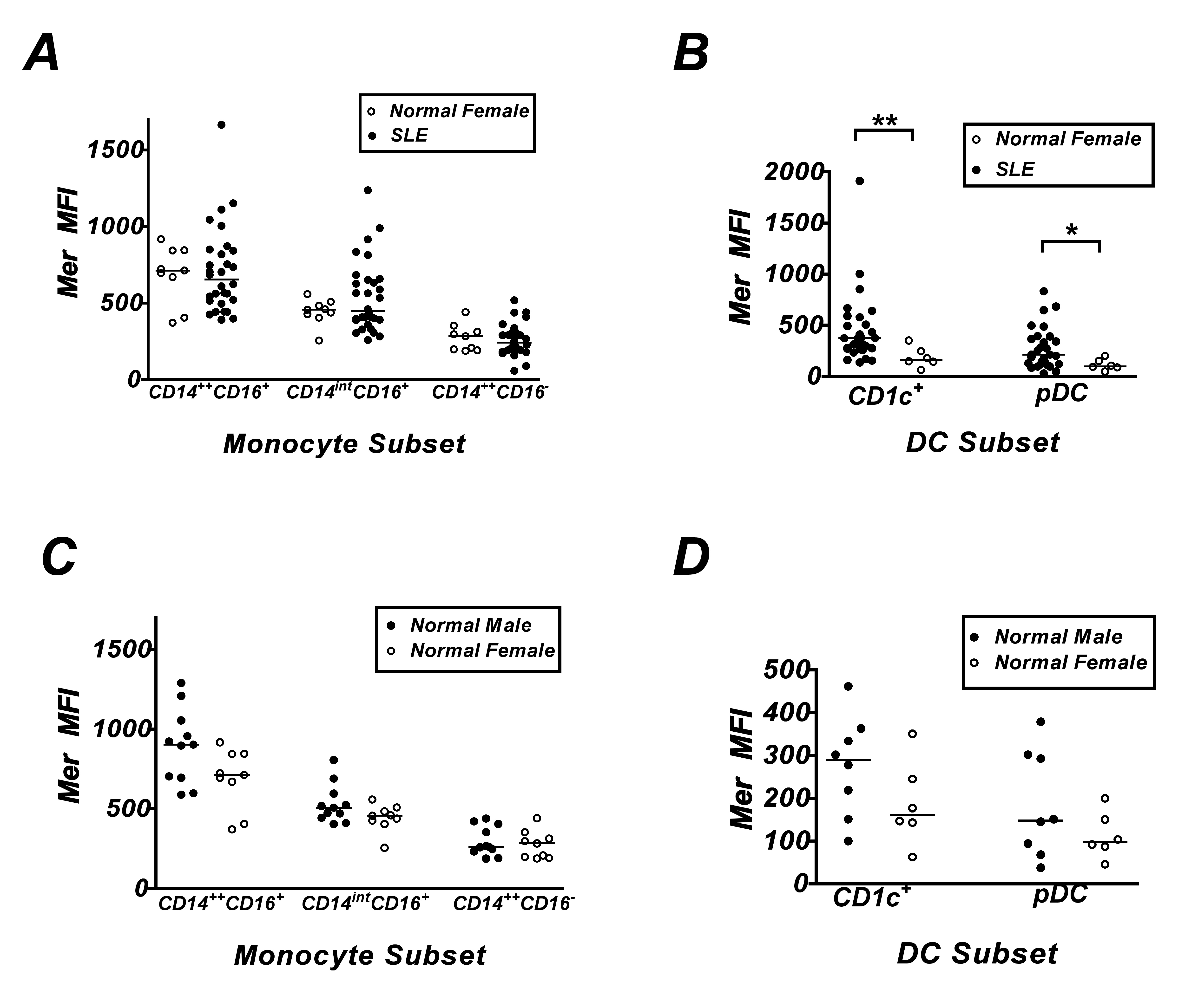


The Mann-Whitney U test was used to determine significance. Horizontal bars represent median values in all graphs. ** p<0.001, * p<0.01.

**Additional File 1 Figure S3**

A

Monocyte Subset Proportions


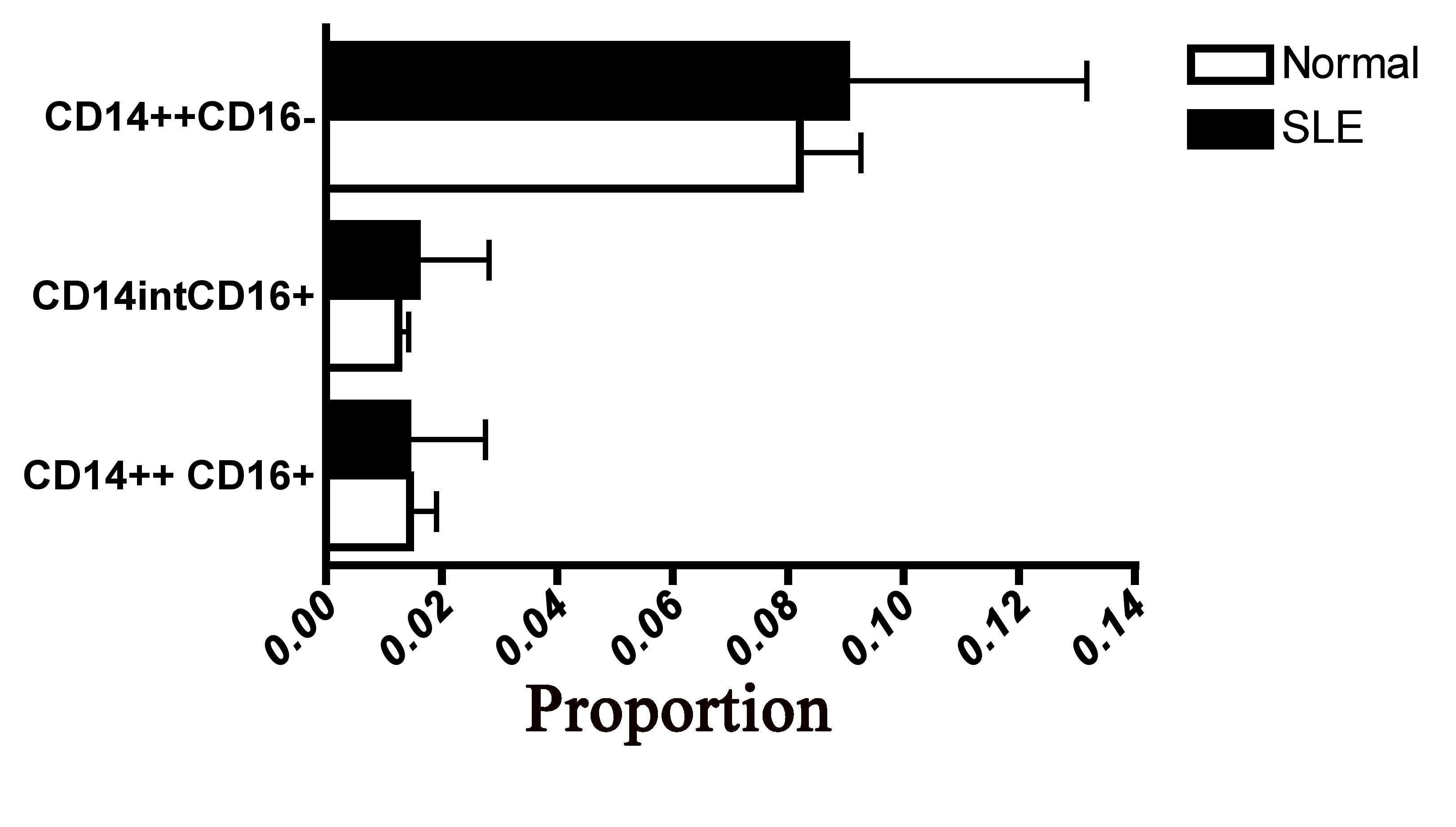


B


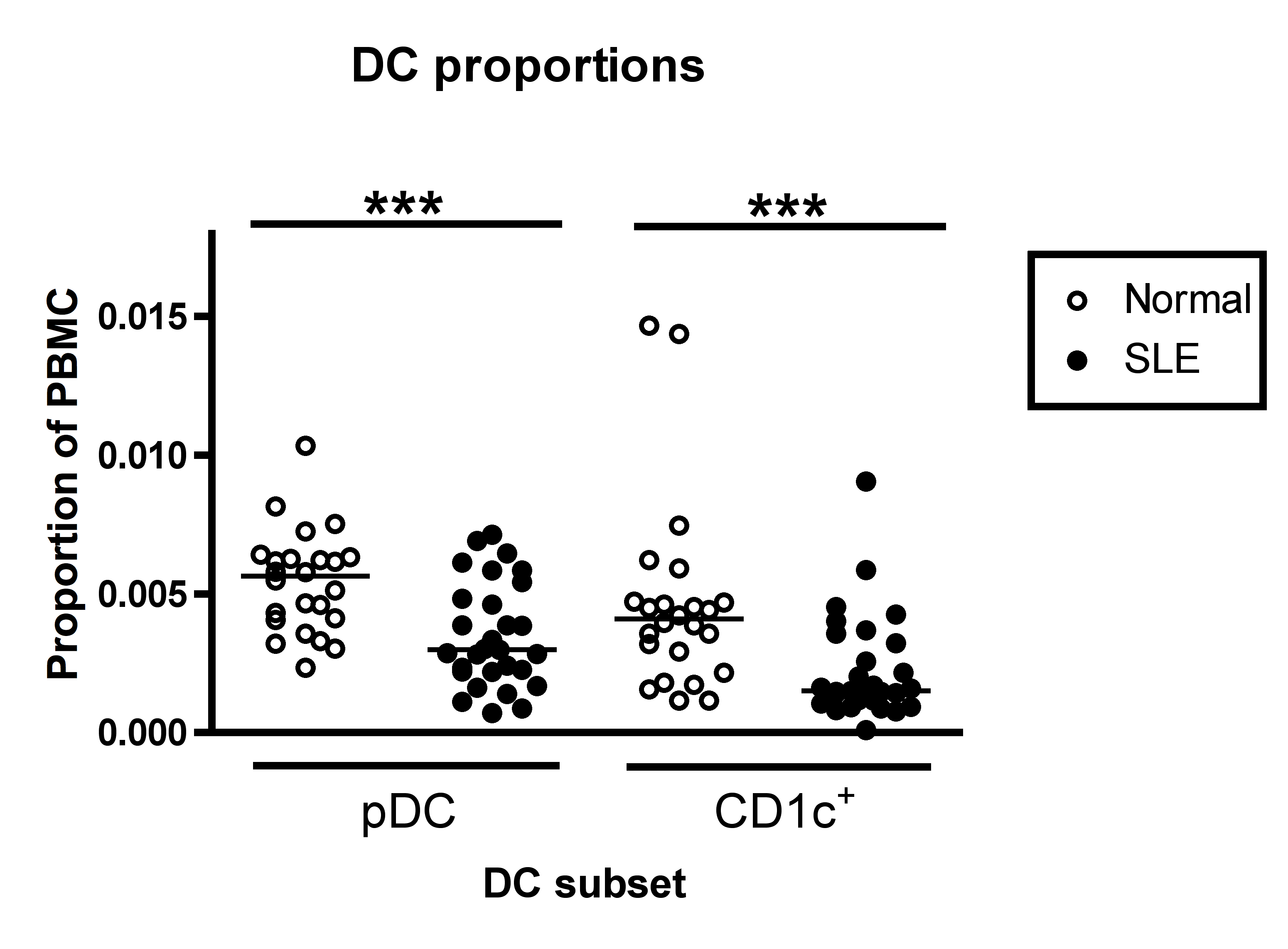


The horizontal lines represent the medians and the Mann-Whitney U test was used to test the significance of the data. *** p ≤ 0.001.

**Additional File 1 Figure S4**





Spearman’s rank correlation was used to determine significance. Values for Spearman’s r and p value are given for the correlations.

**Additional File 1 Figure S5**

**Additional file 1 figure legends.**

**Additional File 1 Figure S1**: Mer and Tyro3 expression in cell lines. **A)** Cell lines U937, Phorbol myristate acetate stimulated THP-1, and Jurkat stained with monoclonal anti-Mer-PE. **B)** K562 cells stained with monoclonal anti-Tyro3-PE

**Additional File 1 Figure S2.** Comparison of Mer and Tyro3 expression in leukocyte populations in the blood of normal healthy subjects and SLE patients. **A)** Expression on Mer on lymphocyte populations in normal individuals and patients with SLE. **B)** Expression of Tyro3 on leukocyte subpopulations from normal individuals and patients with SLE.

**Additional File 1 Figure S3**. Comparison of Mer expression in normal females with SLE patients and effect of gender on expression of Mer. **A)** Mer expression levels on monocytes from normal female and patients with SLE. **B)** Mer expression levels on dendritic cells from normal female and patients with SLE. **C)** Mer expression levels on monocytes from normal female and male subjects. **D)** Mer expression levels on dendritic cells from normal female and male subjects.

**Additional File 1 Figure S4**. Comparison of monocyte and dendritic cells proportions in normal healthy subjects and SLE patients. **A)** Monocyte population proportions of peripheral blood mononuclear cells are similar between SLE and normal healthy subjects. The bars represent the mean values. **B)** CD1c^+^ and plasmacytoid dendritic cell proportions of peripheral blood mononuclear cells are reduced in patients with SLE compared to normal control subjects.

**Additional File 1 Figure S5**. Mer expression on monocytes, sMer in blood and proportions of monocyte subsets correlate with IFN I activity in SLE patients that do not receive prednisone.

Mer levels on monocyte subsets **A)** CD14^++^CD16^+^, **B)** CD14^int^CD16^+^, and **C)** CD14^++^CD16^-^, and sMer levels in **D)** plasma, positively correlate with interferon activity. Proportions of monocyte subsets correlate with IFN I activity, negatively for **E)** CD14^int^CD16^+^ monocytes, and positively for **F)** CD14^++^CD16^-^ monocytes.
